# The NLRP3 inflammasome: a new player in neurological diseases

**DOI:** 10.3906/biy-1909-31

**Published:** 2019-12-13

**Authors:** Elif EREN, Nesrin ÖZÖREN

**Affiliations:** 1 Department of Molecular Biology and Genetics, Apoptosis and Cancer Immunology Laboratory (AKIL), Boğaziçi University, İstanbul Turkey; 2 Center for Life Sciences and Technologies, Boğaziçi University, İstanbul Turkey

**Keywords:** NLRP3, inflammasomes, Alzheimer’s disease, Parkinson’s disease

## Abstract

Inflammasomes are supramolecular protein complexes implicated in the detection of pathogens or danger-associated molecules and are responsible for mounting the first line of innate immune response to counteract these signals and restore tissue homeostasis. Among different inflammasomes identified so far, NLRP3 is of main interest since mutations in Nlrp3 gene are associated with autoinflammatory diseases such as Muckle–Wells syndrome, neonatal onset multisystem inflammatory disease, and familial cold urticaria/autoinflammatory syndrome. On the other hand, whereas other inflammasomes are mainly detectors of specific molecular motifs, NLRP3 is acting as a general sensor of cellular perturbations including potassium efflux, lysosomal damage, and ROS production. Besides this central role of NLRP3 in inflammation, recent publications show that the NLRP3 inflammasome is also involved in the physiopathology of several neurological disorders including Alzheimer’s disease, Parkinson’s disease, and multiple sclerosis. This review gives an overview of the established functions of the NLRP3 inflammasome in mediating inflammation in macrophages and describes its recently discovered roles in neurological disorders in promoting neuroinflammation, as well as modulating key proteins mediating the disorders. Finally, we discuss the targeting of NLRP3 in neurological diseases and present some examples of NLRP3 inhibitors that could be used in neurological disorder treatments.

## 1. Introduction 

### 1.1. The NLRP3 inflammasome

Inflammasomes are cytosolic multiprotein complexes mainly expressed in immune cells and are responsible for the detection and elimination of pathogens or pathogen-associated molecules as well as danger-associated molecular patterns (Martinon et al., 2002). Several inflammasomes activated by specific ligands have been described so far: NLRP3, Pyrin, NLRP1, NLRC4, and AIM2 are induced, among other stimulants, by ATP (Mariathasan et al., 2006), *Clostridium difficile *(Xu et al., 2014), muramyl dipeptide (Hsu et al., 2008), bacterial flagellin (Sutterwala et al., 2007), and polydA:dT (Hornung et al., 2009), respectively.

Induction of these inflammasomes triggers the proteolytic cleavage of caspase-1 that in turn cleaves the proforms of IL-1β, IL-18, and gasdermin D into their active mature forms. The mature N-terminal domain of gasdermin D is inserted into the plasma membrane to form pores and triggers a specific immunological cell death called pyroptosis (Shi et al., 2015; Liu et al., 2016). Mature IL-1β is then secreted from the cells through these gasdermin D pores into the extracellular matrix to recruit other immune cells to the infection site in order to eliminate the trigger and reestablish tissue homeostasis (He et al., 2015; Jorgensen et al., 2016).

**Figure 1:**
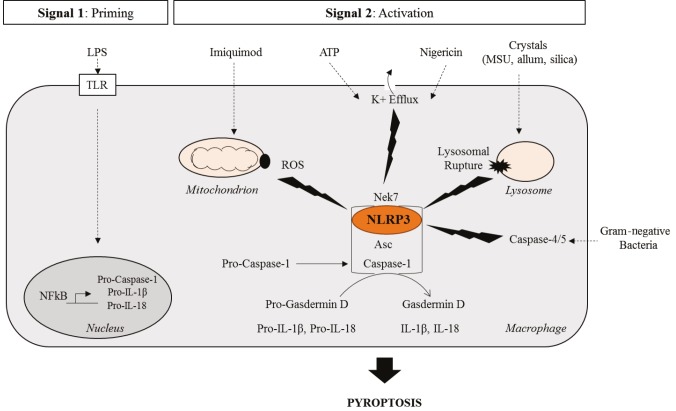
Activation mechanisms of the NLRP3 inflammasome. The NLRP3 inflammasome requires two signals to be activated: the priming signal conferred by stimulation of TLRs by LPS and subsequent induction of inflammasome components’ gene expression through the NFkB pathway and secondly, an activatory signal that triggers the oligomerization of the complex NLRP3 with ASC and Caspase-1; that could be either ROS production by the mitochondria induced by Imiquimod, potassium efflux resulting from ATP or Nigericin stimulation or lysosomal rupture triggered by phagocytosed crystals. The NLRP3 inflammasome is also indirectly activated through the noncanonical inflammasome formed by caspase-4/5 that senses lipopolysaccharide from gram-negative bacteria. LPS: Lipopolysaccharide; TLR: Toll-like receptor; ROS: Recative oxygen species; ATP: Adenosine triphosphate

NLRP3 protein is a member of NOD-like receptors (NLRs) composed of an N-terminal pyrin providing a homotypic protein/protein interaction, a central NACHT responsible for oligomerization, and a C-terminal leucine rich repeat (LRR) domain which may act as a “sensor” and inhibit NLRP3 by its folding on the complex. Upon its stimulation by ion flux triggering molecules such as ATP and nigericin or monosodium urate crystals (MSU), NLRP3 forms a supramolecular complex with the adaptor protein ASC that in turn recruits the effector protease caspase-1 to constitute a multiprotein complex, otherwise known as “specks”, that serves as a platform for the nucleation of the inflammasome (Sehlik et al., 2003). However, unlike other known inflammasomes, the NLRP3 inflammasome requires two signals to be induced: the priming and the activation (Figure 1). First of all, cells need to be primed by the activation of membrane-bound toll-like receptors (TLRs) to induce the expression of the inflammasome components NLRP3, caspase-1, and pro-IL-1β through the NFκB pathway or type I IFN pathway, in the case of endosomal TLRs (Bauernfeind et al., 2009). This priming step is generally induced through the stimulation of TLR4 by lipopolysaccharide (LPS). This ligand triggers a cascade of signaling that results in the induction of inflammasome components’ gene expression.

Once primed, a second signal is required to fully activate the complex. To date, three different events have been found to provide this signal: lysosomal destabilization and rupture (Hornung et al., 2008), mitochondrial reactive oxygen species (ROS) production (Zhou et al., 2011), and decrease of intracellular potassium concentration (Petrilli et al., 2007; Muñoz-Planillo et al., 2013). When crystals such as monosodium urate or aluminum are phagocytosed by cells, because of their size and their structure, they induce lysosomal homeostasis dysregulation, which may result in lysosomal rupture. The release of several components that are supposed to be confined into these organelles, for instance cathepsin G, activates the NLRP3 inflammasome. Similarly, dysregulation of mitochondrial metabolic activity through the impairment of voltage-dependent anion channel is sensed by NLRP3 that directly binds to cardiolipin that is a lipid component of the inner membrane of mitochondria (Zhou et al., 2011; Iyer et al., 2013). Finally, changes in ionic (Ca++, K+) flux triggered by the stimulation of P2RX7 receptors by nigericin or ATP cause the efflux of potassium that is sensed by NLRP3, that gets activated.

Although the activation of the NLRP3 inflammasome is tightly regulated at different levels, mutations in the *Nlrp3* gene resulting in overactivation of the complex were identified in patients with autoinflammatory diseases (Aganna et al., 2002). For this reason, to cure these diseases NLRP3 inflammasome targeting strategies were developed. Among the most efficient is Anakinra treatment; Anakinra is a molecule that antagonizes the IL-1 receptor, thus blocking IL-1 signaling triggered by excessive IL-1β secretion was observed in these patients (Hawkins et al., 2004). Another possible treatment is the use of the specific NLRP3 inhibitor MCC950 (Coll et al., 2015). In a mouse model of Muckle–Wells syndrome induced by *Nlrp3* mutation, administration of MCC950 increased mice’s survival and diminished IL-18 levels in the serum (Coll et al., 2015). However, contradictory findings showing that MCC950 is only effective on the inhibition of WT NLRP3 also exist (Wall et al., 2019 BioRxiv). Nonetheless, as it will be described below, inhibition of NLRP3 by MCC950 in pathological conditions involving nonmutant NLRP3 is still under investigation in neurological disorders and will probably enter clinical trials soon. Despite these extensive studies on NLRP3, our knowledge on this inflammasome is mainly limited with its functions in macrophages. In fact, recent publications suggest a new role for NLRP3 in the context of neurological disorders. 

### 1.2. Neuroinflammation 

Microglial cells are the immune cells of the central nervous system that are considered tissue-resident macrophages responsible for preserving brain homeostasis to provide an adequate environment for the neurons to function. Microglial cells express many pathogen recognition receptors, including NLRP3, that allow their activation in response to pathogen infiltration through the blood brain barrier or in the case of injuries. Microglia are highly active cells that survey the brain and when activated are able to phagocytose and eliminate abnormal protein deposits in the brain seen in some neurological diseases and to secrete chemokines to increase the blood brain barrier permeability promoting the recruitment of other lymphocytes to the infection/injury site (Nimmerjahn et al., 2005). However, the protective neuroinflammation triggered by microglial cells can become detrimental for the host in certain pathological conditions. Pathological neuroinflammation is caused by abnormally high cytokine/chemokine secretion due to an excessive amount of stimulants (Alzheimer’s and Parkinson’s diseases), infection (meningitis) or physical or mechanical injuries (traumatic brain injuries), and vascular occlusions resulting in an excessive inflammasome activation, dysregulation of blood brain barrier (BBB) permeability or BBB breakdown, and increased infiltration of peripheral immune cells. In the following section, different examples of neurological disorders will be given and the role of the NLRP3 inflammasome in the development of these diseases will be presented. 

## 2. NLRP3 in neurological diseases

**Figure 2:**
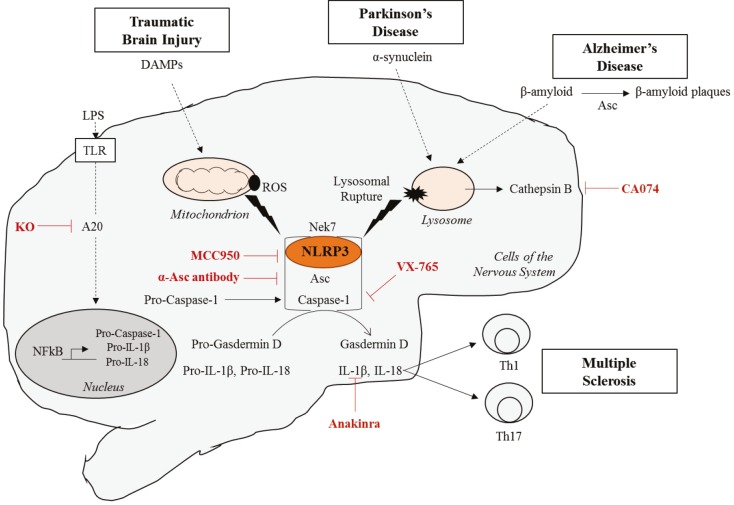
The NLRP3 inflammasome in neurological disorders and novel inhibitor drugs. The NLRP3 inflammasome is activated in reponse to several neurological disorder-triggering molecules. While β-amyloid and α-synuclein, implicated in Alzheimer’s and Parkinson’s disorders respectively, induce lysosomal rupture and Cathepsin B-dependent NLRP3 inflammasome activation; danger associated molecular patterns (or DAMPs) released during traumatic brain injury trigger ROS-dependent NLRP3 inflammasome activation. Different inhibitory molecules of the NLRP3 inflammasome that resulted in the improvement in the disease outcome are also shown.

Neuroinflammation is a driving force of the physiopathology of several neurological diseases. These patients present in their plasma or cerebrospinal fluid an increased level of IL-1 family cytokines IL-1β and IL-18 that are controlled by inflammasomes. The involvement of the NLRP3 inflammasome in Alzheimer’s disease, Parkinson’s disease, multiple sclerosis, and traumatic brain injury will be presented (Table; Figure 2).

**Table:** The NLRP3 inflammasome in neurological disorders.

	Species	Molecular mechanisms of NLRP3 activation	References
Alzheimer disease	Human	Elevated IL-1β levels in the cerebrospinal fluid of AD patients.	Halle et al., 2008
	Mouse	Aβ is phagocytosed by mouse microglial cells and induce lysosomal rupture and cathepsin B release.	Halle et al., 2008
		In APP/PS1 model of AD, depletion of Nlrp3 improves memory, decreased IL-1β levels and reduced Aβ deposits.	Heneka et al., 2013
		ASC specks secreted by Nlrp3-activated microglia promotes the oligomerization of Aβ and increase plaque formation.	Venegas et al, 2017
		Tau-dependent neurofibrillary tangles formation was reduced by ASC targeting.	Stancu et al., 2019
Parkinson disease	Human	α-synuclein is cleaved by caspase-1 and in a neuronal cell line α-synuclein aggregation is prevented by caspase-1 inhibition.	Wang et al., 2016
		Costaining of α-synuclein and caspase-1 in the postmortem brain of PD patients.	Wang et al., 2016
	Mouse	Fyn and CD36-dependent α-synuclein phagocytosis and ROS production leading to NLRP3 activation.	Panicker et al., 2019
		In MPTP-induced mouse model of PD, depletion of Nlrp3 or blocking IL1β receptor ameliorated the PD phenotype.	Lee et al., 2019
Traumatic brain injury	Mouse	Increased caspase-1 and IL-1β cleavage and secretion.	Xu et al., 2018
Multiple sclerosis	Mouse	In experimental autoimmune encephalomyelitis (EAE) improvement of phenotype by Nlrp3 depletion.	Gris et al., 2010

### 2.1. Alzheimer’s disease

Alzheimer’s disease (AD) is a sporadic late onset neuropathology characterized by the accumulation of an insoluble form of amyloid****β (Aβ) proteins in the brain (Masters et al., 2015). Aβ is generated by the cleavage of amyloid precursor protein (APP) at different sites by Presenitin-1 (PS1) and Presenitin-2 (PS2) enzymes from the secretase family. Whereas cleaved forms of Aβ are soluble and have short half-life in the brain of healthy individuals through its degradation by specific enzymes or phagocytosis by microglia; in AD patients, a 42 kDa insoluble form is predominant and accumulates to form Aβ plaque deposits in the extracellular milieu. This in turn triggers the accumulation of the tau protein that leads to the formation of neurofibrillary tangles in neurons. Altogether, these phenomena result in synaptic loss, neuronal death, and dysregulated neurotransmitter signaling (Masters et al., 2015). 

Amyloid β proteins, the molecular hallmark of AD, are phagocytized by microglia and induce the secretion of IL-1β in cerebrospinal fluid of patients. Fibrillar but not soluble Aβ was found to induce the NLRP3 inflammasome in a mouse microglial cell line in a phagocytosis-dependent manner (Halle et al., 2008). Phagocytized Aβ accumulated in the lysosomes and triggered lysosomal destabilization and rupture and cathepsin B release into the cytosol (Halle et al., 2008), consistent with the fact that higher amount of cathepsin B is found in Aβ plaques and cathepsin B inhibition ameliorates AD. Lysosomal damage induced by Aβ internalization triggered NLRP3 activation, ASC speck formation, caspase-1 maturation, and IL-1β secretion.

 Another evidence for the involvement of the NLRP3 inflammasome in AD pathology comes from the studies of AD-associated mutant APP/PS1 expressing mice that are also Nlrp3 knocked out (APP/PS1/Nlrp3-/-; Heneka et al., 2013). In fact, while Aβ accumulated in the brain of APP/PS1 mice and triggered AD symptoms, impairment of the *Nlrp3* gene in these mice ameliorated their AD phenotype: APP/PS1/*Nlrp3*-/- mice have decreased IL-1β levels in total brain, absence of caspase-1 cleavage and improved memory test. Moreover, depletion of *Nlrp3* considerably reduced the amount of Aβ plaque deposits in the brain of APP/PS1/*Nlrp3*-/- compared to APP/PS1 mice and differentiated microglia towards a M2 type antiinflammatory immune response favoring tissue remodeling and Aβ clearance (Heneka et al., 2013).

In addition, ASC specks formed in response to NLRP3 activation have some prionic activity as they are secreted from the cells and induce the oligomerization of other proteins (Franklin et al., 2014). Similarly, in AD, ASC specks are secreted from microglia, bind to Aβ, increase their oligomerization, and facilitate their propagation in different areas of the brain (Venegas et al, 2017). *Asc* gene knockout, antibody targeting, or mutation of pyrin domain of ASC responsible for oligomerization significantly reduced Aβ deposit and ameliorated cognitive functions of AD model of mice (Venegas et al., 2017).

Besides amyloid plaques, neurofibrillary tangles are also observed in the brain of AD patients as a result of tau protein accumulation. Tau protein was shown to activate the NLRP3 inflammasome in microglia and its oligomerization was exacerbated by ASC similar to Aβ plaques (Stancu et al., 2019). Depletion of *Asc *or inhibition of the NLRP3 inflammasome by MCC950 reduces tau-induced neurofibrillary tangle formation (Stancu et al., 2019). 

### 2.2. Parkinson’s disease

Parkinson’s disease (PD) is a neurological disease causing the death of dopaminergic neurons thus impairing the movement of patients. PD is characterized by the accumulation of α-synuclein protein in the cytoplasm of neurons (Poewe et al., 2017). α-synuclein is encoded by the *SNCA* gene and is normally degraded by the ubiquitin/proteasome or lysosomal autophagy system. However, in Parkinson’s disease, mutations in critical players of the lysosomal pathway such as *LRRK2*, *GBA* genes were identified. α-synuclein forms protofibrils and then fibrils that will finally constitute the major part of Lewis bodies and cause neuron loss and dopamine deficiency. The signal is amplified as dysregulation of the lysosomal pathway leads to synuclein accumulation whereas these aggregates in turn inhibit lysosomal pathway functioning (Poewe et al., 2017).

Interestingly, activation of the NLRP3 inflammasome in a neuronal cell line resulted in α-synuclein aggregation that could be prevented by caspase-1 enzymatic activity inhibition by using VX765 or knockdown using shRNA. Caspase-1 was able to directly cleave α-synuclein at Asp121 in in vitro**experiments and this cleaved form was more prone to aggregation than the others (Wang et al., 2016). Indeed, Lewis bodies isolated from postmortem PD patients showed costaining of caspase-1 and α-synuclein in these structures suggesting an important role of caspase-1 in PD pathology (Wang et al., 2016). Similarly, uptake of α-synuclein by microglia was mediated by Fyn and CD36 and led to NLRP3 inflammasome priming and activation in these cells due to nuclear translocation of NFκb and to mitochondrial malfunction resulting in reactive oxygen species generation (Panicker et al., 2019).

Moreover, depletion of *Nlrp3* protected from dopaminergic neuron loss, and decreased microglia recruitment and activation and resulted in a lower level of IL-1β in a mouse model of 1-methyl-4-phenyl-1,2,3,6-tetrahydropyridine (MPTP)-induced mouse model of PD (Lee et al., 2019). Stimulation of primary microglia cells with MPTP in vitro**triggered NLRP3-dependent caspase-1 and IL-1β maturation as well as increasing pro-IL-1β levels. MPTP induced neuroblastoma cell lines death in a NLRP3-dependent manner and PD phenotype could be reverted in MPTP-mice by IL-1β receptor antagonist administration (Lee et al., 2019).

### 2.3. NLRP3 in other neurological disorders

NLRP3 is also implicated in other neurological conditions such as multiple sclerosis and traumatic brain injury. Multiple sclerosis is a chronic inflammatory disease characterized by T cell reactivity to self that causes demyelination of the neurons and appearance of brain lesions. These lesions trigger the infiltration of immune cells and the breakdown of the blood brain barrier in some cases resulting in excessive inflammation in the brain (Filippi et al., 2018). Extensive studies were performed in mice in a model of experimental autoimmune encephalomyelitis (EAE). Increased NLRP3 levels were observed in EAE model and *Nlrp3*-/- showed less immune cell infiltration, less demyelination and improved neurological scores. NLRP3 was activated in multiple sclerosis and induced the secretion of IL-1β as well as IL-18. IL-18 in turn activates IFN-γ and IL-17 production thus eliciting a Th1 and Th17 immune response that was impaired in *Nlrp3*-/- mice (Gris et al., 2010). These data suggest that NLRP3 not only has a role in the innate immune response mediated neuroinflammation but also modulates adaptive response by regulating T-cells. On the other hand, physical and/or mechanical damage in the brain result in traumatic brain injury (Blennow et al., 2016). Danger-associated molecular patterns released following these injuries lead to NLRP3 activation and neuroinflammation. Increased NLRP3 and cleaved caspase-1 and IL-1β levels were found in mouse models of traumatic brain injuries suggesting an important role for NLRP3 inflammasome (Xu et al., 2018).

## 3. Other inflammasomes in neurological diseases

Besides the NLRP3 inflammasome, NLRC4 inflammasome was also shown to be implicated in neurological diseases. Lysophosphatidylcholine (LPC) that accumulates in multiple sclerosis and Alzheimer’s disease (Farooqui et al., 2006) was shown to activate both the NLRP3 and NLRC4 inflammasomes and to result in the increased IL-1β secretion seen in these patients (Freeman et al., 2017). Moreover, the NLRC4 and NLRP3 inflammasomes were also found to play a role in glioma that is a tumor of the central nervous system. Whereas both NLRP3 and NLRC4 were expressed in the brain and colocalized with caspase-1, only NLRC4’s expression was elevated in glioma samples and correlated with poor diagnostic of these patients (Lim et al., 2019).

Not only NLRP3 and NLRC4 but also NLRP1 inflammasome were shown to have a role in neurological diseases. Polymorphisms in the *NLRP1* gene are associated with Alzheimer’s disease (Pontillo et al., 2012) and multiple sclerosis (Maver et al., 2017), the latter resulting in an increase of IL-1β expression in peripheral blood mononuclear cells isolated from patients carrying these polymorphisms. Moreover, increased NLRP1 expression was measured in monocytes isolated from Alzheimer’s disease patients upon LPS and amyloid beta stimulation (Saresella et al., 2016).

Finally, the DNA sensing AIM2 inflammasome was shown to have a deleterious role in experimentally induced ischemic brain injury by middle cerebral artery occlusion since knockout mice presented improved neurological outcomes and reduced inflammation (Denes et al., 2015). AIM2 levels were upregulated in spinal cord ischemia reperfusion after traumatic injury and targeting AIM2 prevented neuronal pyroptosis (Li et al., 2019). Altogether, these suggest that several inflammasomes, mostly in cooperation with NLRP3 or occasionally alone, have an important role in the physiopathology of neurological disorders. However, for most of them, the stimulant that activates these inflammasomes remains unclear.

## 4. Inhibiting NLRP3 in neurological diseases

### 4.1. MCC950 

Being of central interest due to its involvement in autoinflammatory diseases, extensive searches have been conducted to identify an NLRP3 inflammasome inhibitor (Figure 2). The diarylsulfonylurea compound MCC950/Cytokine release Inhibitory Drug 3 (CRID3) is a small molecule firstly identified to inhibit IL-1β secretion. Further studies demonstrated that MCC950 interferes with ASC oligomerization, caspase-1 cleavage, IL-1β secretion and pyroptosis (measured by LDH release) in response to NLRP3 stimulation, with no effect on other inflammasomes or caspase-11-induced cell death (Coll et al., 2015). MCC950 is a reversible inhibitor that directly binds to both active and inactive NLRP3’s NACHT domain responsible for oligomerization and inhibits the hydrolysis of ATP to ADP necessary for oligomerization (Coll et al., 2019). MCC950 was shown to maintain NLRP3 in its closed inactive state upon its binding preventing then its oligomerization and activation (Tapia-Abellán et al., 2019).

Several in vivo experiments were performed to test the efficacy of MCC950 in neurological diseases. Treatment of mice with MCC950 ameliorated their clinical score of mice and reduced in the number of cells producing IFN-γ and IL-17 in a mouse model of EAE (Coll et al., 2015). In several other studies, MCC950 was found to efficiently repress the NLRP3 inflammasome induced by traumatic brain injury and decrease the neurological severity score in mice model of unilateral cortical impact, controlled cortical injury impact and isofluorane-induced traumatic brain injuries (Fan et al., 2018; Ismael et al., 2018; Xu et al., 2018). 

In a mouse model of APP/PS1 mice, besides inhibiting NLRP3-dependent symptoms seen in Alzheimer’s disease, MCC950 also increased the uptake of β-amyloid plaques and ameliorated neurological outcomes by inducing their clearance (Dempsey et al., 2017). Finally, inhibition of NLRP3 by MCC950 was also investigated in the context of Parkinson’s disease. NLRP3 and ASC levels were upregulated and NLRP3, ASC, and cleaved caspase-1 colocalized with Iba the marker for activated glia in postmortem brain sections of PD patients as well as two mouse models of PD triggered by either 6-hydroxydopamine or α-synuclein performed fibril injections (Gordon et al., 2018). Oral administration of MCC950 efficiently blocked cleaved caspase-1 generation in the brain in vivo in α-synuclein performed fibril induced-, 6-hydroxydopamine induced-PD, and MitoPark mouse model of PD (involving the knockout of *Tfam* gene and resulting in mitochondrial respiratory chain deficiency). MCC950 also decreased dopaminergic neuron death 6-hydroxydopamine induced-PD model and α-synuclein performed fibril-PD. Moreover, chronic administration of MCC950 decreased accumulation of α-synuclein without affecting protein levels. Altogether, these findings make the NLRP3 inhibitor MCC950 a good therapeutic for Parkinson’s disease (Gordon et al., 2018). MCC950 is predicted to enter human clinical trials in 2020.

### 4.2. Caspase-1 inhibition 

Caspase-1 is a central player in the activation of the NLRP3 inflammasome. It is the effector that activates IL-1β/IL-18 and gasdermin D that mediates the immunological outcomes. Before being found to be a component of inflammasomes, caspase-1 was identified as “the interleukin 1- converting enzyme” and many inhibitor molecules have been designed and tested to inhibit caspase-1’s activity (Kostura et al., 1989). In the context of neurological diseases, caspase-1 has a deleterious role in the disease progression since caspase-1 knockout mice were protected from clinical hallmarks of Alzheimer’s disease (Flores et al., 2018). Administration of a reversible small caspase-1 specific inhibitor VX-765 in Alzheimer’s disease model of mice showed improvement of mice clinical behaviors, resulted in decrease in hippocampal and cortical IL-1β levels and prevented amyloid beta deposit (Flores et al., 2018). VX-765 also called Belnacasan is an orally active form of VRT-043198 and although being promising in the eradication of Alzheimer’s disease manifestations, the necessity of continuous administration of VX-765 and treatment at very early stages of the disease, VX-765 seems to stay as a proof-of-concept of caspase-1’s implication in Alzheimer’s disease. 

VX-765 was also used in the inhibition of Caspase-1 in a mouse model of MS. Increased IL-1β, IL-18, caspase-1, and gasdermin D levels and MS lesions positive for gasdermin D staining were found in postmortem samples of MS patients and EAE model (McKenzie et al., 2018). Treatment of EAE mice with caspase-1 inhibitor VX-765 decreased upregulated transcript levels, reduced gasdermin D stainings, and restored microglia density suggesting an improvement of the disease (McKenzie et al., 2018). Even if not frequently involved, the presence of caspase-1-independent pathways leading to IL-1β maturation and the possibility of triggering gasdermin D-mediated pyroptosis by caspase-11, force researchers to find an alternative way of targeting NLRP3.

### 4.3. A20 

The priming by NFκB pathway is an essential step in the activation of the NLRP3 inflammasome. The A20 protein negatively regulates this pathway by acting on its players through the modulation of the ubiquitination resulting in either the destabilization of protein/protein interactions or the degradation of these proteins (Coornaert et al., 2009). Mutations in *Tnfaip3* gene encoding for A20 were associated with increased susceptibility to autoinflammatory diseases (Ma et al., 2012). A20-deficient mice developed spontaneous neuroinflammation and promoted axon injury (Guedes et al., 2014). Interestingly, A20 is highly expressed in developing and mature adult nervous system (Voet et al., 2018). As full knockout of A20 led to massive inflammation and organ failure, conditional A20 KO mice were generated to investigate the effect of A20 in the brain. The number of microglia was increased in the brain and cerebrospinal fluid of conditional knockout mice and proinflammatory genes were upregulated in these cells. As a result, A20 KO mice showed poor cognitive function. A20 was also protective against LPS injections and EAE model of MS. A20 mediated its protective effect through the inhibition of the NLRP3 inflammasome as *Nlrp3*/A20 double knockout mice showed a better clinical score than A20 knockout mice in EAE (Voet et al., 2018). 

### 4.4. Inhibitory NLR proteins

NOD-like receptors (NLRs) are a family of cytosolic proteins that besides forming inflammasome complex (such as NLRP1, NLRP3, NLRP6, NLRC4), can also have antiinflammatory roles. NLRP10, NLRX1, and NLRC3 proteins belong to the last group. These proteins were found to negatively regulate NFκB pathway as well as NLRP3 inflammasome itself. NLRX1 was first identified as an inhibitor of the NFκB pathway (Allen et al., 2011) and a modulator of the RIG-I/MAVS pathway during viral infections (Allen et al., 2011; Moore et al., 2008). Besides this antiinflammatory role, NLRX1 is also neuroprotective in mouse model of multiple sclerosis (Eitas et al., 2014). *Nlrx1*-/- mice presented enhanced limb paralysis, more proinflammatory cytokine expression in the spinal cord tissue, increased demyelination and higher immune cell infiltration in the brain suggesting that Nlrx1 is important to control inflammation in the central nervous system and preserve tissue homeostasis in multiple sclerosis (Eitas et al., 2014). Similarly, in a mouse model of controlled cortical impact traumatic brain injury, Nlrx1-deficient mice showed higher brain lesions, worse clinical score, increased inflammation, and higher microglia recruitment and activation (Theus et al., 2017). Moreover, expression analysis in patients with aneurysm revealed that NLRX1 expression is downregulated compared to healthy controls (Theus et al., 2017). In a molecular level, NLRX1 protein regulates glutamate homeostasis, the principal neuro-stimulant amino acid in the central nervous system (Mahmoud et al., 2019). Accumulation of glutamate in the extracellular milieu is toxic for the brain. NLRX1 increases the uptake of glutamate into the astrocyte and inhibits its exocytosis. Although direct inhibition of the NLRP3 inflammasome by NLRX1 was not shown in the brain, it could be a good target since MAVS facilitates oligomerization of NLRP3 and its activation (Park et al., 2013). For instance, in an acute myocardial ischemia, NLRX1 was shown to inhibit NLRP3 inflammasome activation through MAVS expression downregulation (Li et al., 2016).

NLRC3 protein is a CARD domain containing member of the NLR family and was described to have an inhibitory role on NFκB pathway by targeting TRAF6 to ubiquitination (Conti et al., 2009; Schneider et al., 2012). Modulation of the NFκB pathway by NLRC3 was shown to suppress CD4+ T cell activation, thus facilitating *Mycobacterium tuberculosis* escape from the immune system (Hu et al., 2018) or increasing susceptibility to lymphocytic choriomeningitis virus infection (Uchimura et al., 2018). Besides NFκB, NLRC3 also modulates the STING pathway by disrupting STING/TBK1 interaction (Zhang et al., 2014) through direct STING binding and this interaction is disrupted through the binding of viral DNA to the LRR domain of NLRC3 that will liberate STING and activate type I interferon production (Li et al., 2019). Moreover, NLRC3 was found to inhibit specifically NLRP3 inflammasome activation by interfering with the assembly of ASC with caspase-1 thus reducing ASC speck formation caspase-1 maturation of (Eren et al., 2017). Furthermore, in zebrafish, NLRC3 homolog called Nlrc3-like was required for controlling inflammation in the brain. Mutation in Nlrc3-like resulted in increased IL-1β expression in the glia and increased neuronal cell death (Wang et al., 2019). These phenotypes could be reverted by depletion of Asc or inactivation of gasdermin D homolog Gsdmea by morpholino oligos suggesting that Nlrc3-like is an inflammasome inhibitor in zebrafish microglia (Wang et al., 2019). Nlrc3-like was also required for normal microglia development in zebrafish brain (Shiau et al., 2013). In the presence of mutant Nlrc3-like protein, primitive macrophages could not populate the brain and accumulated in the yolk sac with signs of activated macrophages including increased expression of proinflammatory cytokines IL-1β and IL-18 among others. Together with abnormal infiltration of neutrophils in the brain, Nlrc3-like mutation resulted in systemic inflammation. Expression of WT Nlrc3-like rescued the phenotype and molecular analysis revealed that Nlrc3-like directly interacts with Asc thus impairing inflammasome activation (Shiau et al., 2013). The role of Nlrc3 in microglia inflammation or development is not confirmed in mice yet but high expression of NLRC3 in brain and different nervous cells suggest that it may have a similar role in mice and humans. Therefore, expressing NLRC3 could be a good strategy to target both NFκB pathway and NLRP3 inflammasome. Especially, since ASC specks are important vectors of most of the neuropathologies, the ability of NLRC3 to disrupt ASC oligomerization may be benefic for the patients.

## 5. Conclusions

Up to now, NLRP3 inflammasome research was limited to macrophages and especially to mouse bone marrow-derived or human peripheral blood mononuclear cell-derived macrophages or human leukemia cell line THP-1. Recent findings clearly show that NLRP3 inflammasome components are expressed not only in a variety of other cell types including neutrophils, epithelial and endothelial cells but also in some tissue-resident macrophages, such as microglia in the brain, Kupffer cells in the liver, and Langerhans cells in the skin.

Investigation of NLRP3’s role in several neurological disorders reveals that excessive inflammatory signature observed in these pathologies is most often due to increased IL-1β levels in the brain and cerebrospinal fluid of patients. Abnormally folded protein aggregates responsible for the neurodegeneration were either directly generated by inflammasome components, as in the case of the cleavage of α-synuclein by caspase-1 in Parkinson’s disease, or constituted a trigger for NLRP3 activation such as β-amyloid in Alzheimer’s disease. Targeting of the NLRP3 inflammasome at different levels resulted in the amelioration of the disease progression. Interfering with NLRP3 priming by targeting A20 protein, blocking caspase-1 with VX-765 or inhibition of NLRP3 directly with MCC950 gave very promising results. Especially, specific inhibition of NLRP3 by MCC950 by daily oral administration of this molecule allowed disease regression in all mice models of Parkinson’s disease tested. 

Although the majority of studies on neurodegenerative diseases and NLRP3 inflammasome show that NLRP3 inhibition is beneficial for the regression of the disease symptoms, two studies at least suggest that outcomes of NLRP3 activation may have a protective role on the symptoms. For instance, deletion of IL-18, a cytokine that is maturated by caspase-1 cleavage and secreted following NLRP3 activation, increased mortality of APP/PS1 mice as a result of increased deposition of amyloid plaques (Tzeng et al., 2018). A similar protective role of IL-18 was also observed in cerebral ataxia (Andoh et al., 2008). IL-18 is an important regulator of T cell and natural killer cell response. It is clear that neurodegenerative pathologies are quite complex disorders and may involve the participation of both innate and adaptive immune system. Whether neuroinflammation is beneficial or not for the patients is probably determined by several factors including the disease type and the severity of the pathology. 

Another major outcome of the NLRP3 inflammasome activation is the induction of a form of cell death called pyroptosis. Most of the studies are mainly based on caspase-1 and IL-1β levels in patients or mouse model of the diseases. Only in few of them, the role of gasdermin D was studied. Gasdermin D staining in lesions of brain tissues was shown or gasdermin D was found to be responsible for the neuronal death observed. However, other publications suggest that although IL-1 is important, pyroptosis does not have a role in the development of the pathology also exist. Thus, the effect of pyroptosis in neurological disorders has to be further confirmed and if implicated, targeting of gasdermin D in these diseases should also be considered. The relationship between NLRP3 and neurological disorders is an emerging field that needs further investigation. Nonetheless, the targeting of NLRP3 in different neuropathologies is obviously promising.

**Acknowledgements**

Authors would like to thank Boğaziçi University Research Funding via Projects BAP 6526 (11B01D10) and 7360 (13B01M3) to N.Ö.
